# Managing pollution from antibiotics manufacturing: charting actors, incentives and disincentives

**DOI:** 10.1186/s12940-019-0531-1

**Published:** 2019-11-06

**Authors:** Niels Nijsingh, Christian Munthe, D. G. Joakim Larsson

**Affiliations:** 1 Centre for Antibiotic Resistance Research (CARe), at University of Gothenburg, Gothenburg, Sweden; 2 Department of Philosophy, Linguistics and Theory of Science, University of Gothenburg, Gothenburg, Sweden; 3 Institute of Ethics, History and Theory of Medicine, Ludwig Maximilian University, Munich, Germany; 4 Department of Infectious Diseases, Institute of Biomedicine, The Sahlgrenska Academy, University of Gothenburg, Gothenburg, Sweden

**Keywords:** Antimicrobial resistance; management, Environmental pollution, Policy

## Abstract

**Background:**

Emissions of high concentrations of antibiotics from manufacturing sites select for resistant bacteria and may contribute to the emergence of new forms of resistance in pathogens. Many scientists, industry, policy makers and other stakeholders recognize such pollution as an unnecessary and unacceptable risk to global public health. An attempt to assess and reduce such discharges, however, quickly meets with complex realities that need to be understood to identify effective ways to move forward. This paper charts relevant key actor-types, their main stakes and interests, incentives that can motivate them to act to improve the situation, as well as disincentives that may undermine such motivation.

**Methods:**

The actor types and their respective interests have been identified using research literature, publicly available documents, websites, and the knowledge of the authors.

**Results:**

Thirty-three different actor-types were identified, representing e.g. commercial actors, public agencies, states and international institutions. These are in complex ways connected by interests that sometimes may conflict and sometimes pull in the same direction. Some actor types can act to create incentives and disincentives for others in this area.

**Conclusions:**

The analysis demonstrates and clarifies the challenges in addressing industrial emissions of antibiotics, notably the complexity of the relations between different types of actors, their international dependency and the need for transparency. The analysis however also suggests possible ways of initiating incentive-chains to eventually improve the prospects of motivating industry to reduce emissions. High-resource consumer states, especially in multinational cooperation, hold a key position to initiate such chains.

## Background

Antibiotic resistance presents a serious and growing threat to global health. Effective antibiotics constitute not only our most important tool to treat bacterial infections, but are also critical for the effectiveness of many other areas of modern healthcare [1]. Use, misuse and overuse of antibiotics in humans and animals, together with insufficient hygiene and infection control, are the most important drivers of resistance on a global basis. Since bacteria and bacterial genes often move through the environment and across humans and animals [2–4], a ‘One Health’ perspective that takes all three of these domains into account is essential [5–7]. The environment plays a role both in the transmission of resistant pathogens and as a source for resistance factors that over time are transferred horizontally to pathogens. In both cases, antibiotics emitted into the environment create a selection pressure likely to favour resistant strains [8]. Antibiotic pollution occurs, partly, as a result of excretion from humans and domestic animals. Although widespread, it is still uncertain to what extent the levels found from such sources select for resistant bacteria [9]. In contrast, antibiotic pollution due to wastewater emissions from manufacturing plants of antibiotics can be staggering, with concentrations reaching into the mg/L range, constituting strong drivers of resistance [10]. It is therefore not surprising that there is increasing recognition of the need for global coordinated action to reduce industrial antibiotics emissions [4, 11–15].

This paper aims to contribute to the understanding of institutional aspects that is necessary for evidence-based and effective action in this area. We present a map of actors, detailed in the Additional file 1, that may facilitate more effective pollution control and identification of what incentives or disincentives may affect their tendency to act. On this basis, we also address issues about sharing of key responsibilities for initiating and sustaining effective measures to curb industrial antibiotics pollution.

In the next section, we explain the theoretical background, the method we have used when completing the map of actors and incentives, as well as the limitations of our analysis. In the Results section, we use the taxonomy provided in the Additional file 1 and suggest that in answering the question on who should act, we need to distinguish between ‘consumer’ and ‘producer’ countries. By making this distinction, we do not mean to imply that different countries are exclusively producers or consumers of antibiotics, but rather that in analysing the various (dis)incentives, we need to distinguish between different roles of countries as either consumer or producer. We then describe how actors in both antibiotic consumer and producer countries can be motivated to effective action (incentives), as well as factors that would hinder such motivation (disincentives). In the Conclusions section, we summarise the main findings, and expand our analysis to discuss the distribution of responsibilities among the actor types. We conclude that high-income consumer states and certain public institutions within these are in a key position to initiate effective change, especially through multinationally coordinated actions.

## Methods

Any attempt to initiate effective actions to reduce industrial antibiotic pollution has to face intricate realities when trying to decide what actions should be taken, and by whom. The production, trade and consumption chain of pharmaceuticals is complex and involves many actors with different interests. The challenge therefore needs to be approached from a complex system perspective [16]. When considering the costs and benefits of various interventions on specific levels and within specific sectors, we need to also consider how different levels and sectors may interact to either favour or undermine better pollution control. The interdependencies within and between systems and parts of systems of different acting parties therefore need to be understood. This paper aims to take a first step of contributing to such understanding by presenting a map of the relevant types of actors with their respective interests, and describe possible incentives as well as disincentives (some of which already exist, while others may emerge due to policy choices).

The are several proposals in the literature on how to address antibiotics pollution [3, 10–15, 17–20]. Despite providing important contributions, all of the above focus on individual drivers and actors, leaving the systemic perspective largely unexplored. A map of actors and their relations could therefore provide a clearer insight into the ways in which these as well as future proposals may interact and function. The primary intention of the present study is not to argue for or against specific interventions, but to analyse how different types of actions may either support and enhance, or come apart, conflict with or even undermine each other. This will provide some general lines along which actions might be designed and evaluated, and which actors should be thought of as responsible for initiating and sustaining such developments.

Besides peer-reviewed scientific literature, our material has included governmental and corporate websites and policy documents. We thereby identified 33 types of actors, and sorted these into a relational network that illustrates the actors’ main interests, and how they may interact at and in between various levels (local, regional, national, international) in light of these interests. On this basis, we then inventoried the ways in which these actors may be incentivized to act, including regulatory, economic and political incentives.

In doing so, we make no particular assumptions about the underlying motivational patterns of human beings. Specifically, we do not presuppose a rational choice theory nor do we presume that actors are only driven by self-interest, but do assume that institutions act in consideration of the charters, formal aims, and so on, which define and direct them. By focusing on (dis)incentives, we do not mean to deny that actors may be driven by a variety of concerns, among which genuine worries on the development of antibiotic resistance. However, we also do not presume that merely presenting either individual or institutional actors with evidence will be sufficient to prompt them to do what is right from a moral perspective or what desirable from a social perspective: sometimes people need a push to do the right thing. It is an empirical observation that people are often prone to prioritise their short-term interests, which makes pushing the right buttons in the form of incentives of eminent importance in addressing complex, global problems, such as antibiotic pollution. For institutions, such as public agencies and corporations, there is a parallel need to have actions match or adapt to legally mandated goals (such as maximizing shareholder value for limited companies), formal charters and surrounding regulation.

In our analysis, we put to one side driving forces that are not specifically related to properties of the described actors and therefore common to all or many of them, such as inherent tendencies of institutions to work in line with the motivation of their individual employees [21].

The criterion for including actors was that doing so would add to the understanding of how the different incentives and disincentives may come apart or together in relation to the aim of controlling industrial antibiotics pollution. Therefore, we have included a wide set of actor *types*, but did not distinguish between single actors motivated by very similar types of interests, as these are prone to be motivated by the same type of incentives. For instance, we do not distinguish between different national political representatives or functions (as these will likely be motivated by similar mechanisms), or between different public agencies guided by similar statutes and missions, or between different individual pharmaceutical companies of the same type, and so on. We do, however, identify different *types* of political, public, business or non-governmental bodies that seem to act out of relevantly different types of interests – as well as institutions that perform clearly different roles and are thus able to exert influence in different ways.

The outcome of this analysis is a network of actors with attached interests and options to act that may affect the options and/or stakes of at least one other actor. This result is an important step to come closer to a comprehensive prescriptive social network analysis [22, 23]. Moreover, this step already facilitates informed hypotheses of promising policy directions, and analysis of the proper sharing of responsibility to have such policy development initiated. The entire network is mapped and described in more detail in an Additional file 1 to this article. We use examples of specific national actors to serve as illustrations of different national actor types. These have been taken from primarily two contexts well known to us, India and Sweden.

In our conclusions, we also present a qualified generalisation of the results related to these specific examples, noting methodological limitation with regard to details: while in many cases institutions in different countries may perform similar roles, one cannot simply presume that our analysis applies throughout different social and legal contexts. Differences in institutional structure need to be taken into account as this can determine what measures are most effective. In what follows, it will become clear that the properties of producer countries tend to be more easily generalisable than those of the consumer countries, due to the complex institutional structures that are relevant to licensing, sale of drugs, health insurance, hospital care and so on. This limitation therefore mainly affects consumer countries. However, as we will argue, there are nonetheless valuable lessons that apply to all consumer countries and what remains will be important topics to address in future research.

From here on, the presentation refers to the separate Additional file 1 linked to this article, and we will henceforth refer to the actor types detailed in this Additional file 1 by inserting # and the number given to them there in parenthesis.

## Results

The Additional file 1 details 33 separate actor types of potential importance, summarised in Table 1.

**Table 1:** Thirty-three actor types with possibilities to contribute to the reduction of antibiotic emissions from manufacturing. For each actor type, examples of their interests, possible actions and incentives and disincentives for those actions are listed (more details to be found in the main text and the supplementary material). Broadly common interests among actors, e.g. individual’s/employees desire to contribute to positive societal change (reducing pollution, improving public health) are implicit but have not been listed for each actor. For actors #15, 16, 17, 21 and 22 we have listed Swedish examples of actors types

Actors	Interests	Actions	Incentives	Disincentives
# 1 Research-based pharmaceutical companies	- Increase turnover, reduce costs; - Strategic interests (e.g. “stay ahead of the curve); - Reputation concerns; - Preserve effectiveness of product by curbing antibiotic resistance.	- Motivate #3: demand good pollution control for the API’s they buy; - Monitor & reduce their own discharges; - Set internal discharge limits; - Act transparently with regards to production sites (also of suppliers) and environmental performance.	- Emission standards; - Legal requirements; - Economic incentives (price, costs, turnover); - Pressure from investors and buyers; - Reputation concerns.	- Transparency as a threat for reputation concerns; - Higher production cost; - Lack of follow-up of external demands - risks of unfair competition.
#2 Generic pharmaceutical companies	- Increase turnover, reduce costs; - Strategic interests (e.g. “stay ahead of the curve”); - Preserve effectiveness of product by curbing antibiotic resistance.	- Motivate #3: demand good pollution control for the API’s they buy; - Monitor & reduce their own discharges; - Set internal discharge limits; - Act transparently with regards to production sites (also of suppliers) and environmental performance.	- Emission standards; - Legal requirements; - Economic incentives (price, costs, turnover); - Pressure from investors and buyers.	- Higher production cost; - Lack of follow-up of external demands - risks of unfair competition;
# 3 Subcontracting pharmaceutical companies	- Increase turnover, reduce costs; - Strategic interests (e.g. “stay ahead of the curve”); - Preserve effectiveness of product by curbing antibiotic resistance.	- Monitor and reduce their own discharges; - Set internal discharge limits; - Act transparently with regards to environmental performance.	- Emission standards; - Legal requirements; - Economic incentives (price, costs, turnover); - Pressure from investors and buyers (i.e. #1 & #2).	- Higher production cost; - Lack of follow-up of external demands - risks of unfair competition;
# 4 Umbrella organisations/ collaborations between pharmaceutical companies	- Represent members (#1, #2, #3); - Align interests of members.	- Coordinate action	- In addition to those applying to #1,2 and 3: become a stronger force for promoting common interests.	- Interest and priorities may differ between members.
#5 Owners of pharmaceutical companies	- Profit on investment; - Reputation concerns.	- (Threaten to) withdraw investments in #1, #2 and #3; - Power through representation in boards.	- Pressure from customers and interest groups; - Financial incentives (risk for loss of business associated with “scandals”).	- Limits on (short-term) profits, as owners set profit expectations.
# 6 Waste water treatment plants (WWTPs)	- Increase turnover, reduce costs.	- Implement more effective treatment; - Monitor and report emissions.	- Government legislation; - Subsidies.	- Costs.
# 7 Parallel importers	- Increase turnover, reduce costs.	- Promote transparency and regulations.	- Pressure from buyers; - Legislation.	- Very limited ability to gain information on, or to influence the production chain.
# 8 Producing country states	- Represent population; - Protect public health; - Protect economic interests.	- Regulate industry in terms of emissions; - Pressure, negotiate with #1–3; - Sponsor research and knowledge transfer; - Support infrastructure.	- Political pressure from citizens and interest groups; - Treaties, multilateral agreements, foreign pressure; - Public health concerns.	- Economic interests: protecting current industry - strict standards may create disadvantages for national producers; - Lobbying by #1–3.
# 9 Environmental oversight agencies	- Follow statutes and directives as defined by #8; - Protect the environment (and public health)	- Implement and enforce rules and regulations; - Provide data on emissions.	- Pressure from various actors; - Directives deriving from #8.	- Pressure from #1–3, in particular on the local level.
# 10 Citizens of producer states	- Economic concerns; - Public health;- environmental protection.	- Pressure industry and government; - Vote.	- Awareness; - Economic, health and environmental interests.	- Lack of information/ awareness; - Lack of interest; - Lack of effective political power.
# 11 Citizen interest groups, environmental and human rights NGOs	- Represent #10; - Represent particular interests.	- Coordinate action; - Create awareness; - Exert pressure.	- Pressure from supporters; - ‘Mediagenic’ action may be more attractive with an eye on public support.	- Limited political power.
# 12 Inter-governmental political forums (eg. G7)	- Coordinate and represent national and international interests.	- Apply political pressure; - Harmonize policies.	- Input by goverments, political leaders; - Pressure by interest groups, political organisations etc.	- Many different interests, they may not always align.
# 13 United Nations agencies	- Initiate and harmonize collective action on global problems.	- Create awareness; - Harmonize policies across nations; - Exert pressure on industry and governments.	- Pressure from governments, interest groups, political organisations etc.	- Limited power.
#14 Consumer country states	- Represent population; - Protect public health; - Protect economic interests.	- Regulate; - Establish premiums; - Direct research funding; - Direct actions by national agencies; - Influence other consumer states and # 30.	- Political pressure by citizens and interest groups; - Treaties, multilateral agreements, foreign pressure; - (Global) public health concerns.	- Economic interests: costs - Lobbying by #1–3; - Little mass, individually (higher cost to establishing premiums); - Institutional barriers (eg. state generic substitution system).
# 15 National Licensing agencies (*Läkemedelsverket*, LV)	- Follow statutes and directives as defined by #14; - Good, affordable health care.	- Implement standards and licensing of medical products;	- Steering by national government.	- Limited mandate.
# 16 Agencies committed to subsidizing decisions (*Tand- och läkemedelsfömånsverket*, TLV)	- Follow statutes and directives as defined by #14; - Good, affordable health care. - Effective resource allocation.	Potentially (but not currently): - Weigh environmental concerns in reimbursement decisions.	- Steering by national government.	- Limited mandate; - Limited possibilities for action under current statutes.
# 17 Agencies committed to prescription policies (*Socialstyrelsen*, SoS, and *Inspektionen för vård och omsorg*, IVO	- Follow statutes and directives as defined by #14; - Good, affordable health care.	- Issue national treatment guidelines (in cooperation with # 18).	- Steering by national government.	- Limited mandate.
#18 Public health agencies	- Follow statutes and directives as defined by #14; - Good, affordable health care.	- Issue national treatment guidelines (in cooperation with # 17)	- Steering by national government.	- Limited mandate.
#19 Agencies committed to public procurement: *Upphandlingsmyndigheten*	- Follow statutes and directives as defined by #14; - Good, affordable health care.	- Supporting #20, 21 and 22 to put pressure on # 1 and 2	- Steering by national government.	- Limited power.
#20 Public hospitals and clinics	- Follow statutes and directives as defined by #14 and #21; - Represent interests of #26 & #28; - Effective resource allocation.	- Apply environmental criteria in procurement; - Improve awareness.	- Regulation.	- Pressure on cost-efficiency; - Limited negotiating power.
#21 Regional government (county council) and their regional medical products committees (*Läkemedels-kommittér*)	- Represent population; - Protect public health; - Good, affordable health care; - Protect economic interests.	- Steer #20; - Weigh in environmental concerns in regional treatment recommendations.	- Political pressure by citizens and interest groups; - National policies; - Public health concerns.	- Limited power.
#22 Central priority setting organisation for drug procurement (*NT-rådet* & *Sveriges kommuner och landsting*, SKL)	- Effective resource allocation; - Good, affordable health care.	- Help counties act jointly and effectively.	- Steering by national government.	- Limited mandate.
#23 Privately funded and operated clinics and hospitals	- Profit; - Promote and protect health of their patients.	- Apply environmental criteria when buying antibiotics.	- Demands made by subcontracting county councils; - Pressure from #28, 29.	- Very little negotiating power.
#24 Pharmacies	- Profit; - Reputation concerns.	- Take environmental concerns into account when purchasing antibiotics (applicable to some countries, not all); - Improve awareness.	- Media attention; - Attracting costumers.	- Little or no influence over what antibiotics to provide through governmental restrictions (in some countries, but not all).
#25 Insurance companies	- Profit; - Reputation concerns.	- Negotiate, pressure # 1,2.	- Financial considerations (e. g. premiums, or taxes).	- Interest in lower prices.
#26 Physicians and other health care professionals	- Economic interests (in some settings); - Professional ethos.	- Pressure, primarily through #27.	- Increased awareness. - Pressure from lobby groups, particularly #1,2 and 4 (in some settings)	Lack of information/ awareness - Lack of interest/time; - Lack of effective political power.
#27 Physician and other health care professional organisations	Represent interests of #26.	- Pressure relevant policy makers and institutions. - Create awareness among members, the public, politicians and policy makers.	- Pressure by members.	- Lack of effective political power.
#28 Patients/citizens of consumer country states	- Keep costs for medicines low; - Access to antibiotics.	- Support NGO’s; - Vote, exert political pressure; - (When possible) buying “environmentally certified” antibiotics.	- Awareness.	- Lack of information/ awareness; - Lack of interest; - Increased costs for medicines; - Lack of effective political power.
#29 Patient organisations	Represent interests of #28.	- Pressure on county governments or inter-regional coordinating bodies; - Improve awareness.	- Pressure by members;	- Lack of effective political power.
#30 Multinational governing bodies (e.g. the EU)	- Represent member states; - Streamlining the national policies.	- Regulate; - Negotiate, pressure; - Subsidize sustainable practices; - Research funding.	- Political pressure; - Treaties, multilateral agreements, foreign pressure.	- Non-aligning interests between member states; - Lobbying; - Lack of jurisdiction. Outside of e.g. EU.
#31 Agencies of multistate bodies (such as the European Medicines Agency)	- Follow statutes and directives as defined by #14.	- Amend licensing requirements (ERA) to include risks for AMR selection and production emissions; - Include environmental considerations in GMP; - facilitate transparency of production chains.	- Steering by #30.	- Lack of research data to define demands; - Lack of jurisdiction outside of e.g. EU.
#32 Media	- Profit; - Credibility; - Public interest.	- Improve awareness; - Expose polluters; - Demand action from the majority of actors.	- More viewers/readers; - Curiosity; - Increased credibility.	- Opacity of productions chains; - Lack of emission data.
# 33 Scientific researchers and universities	- Reputation; - Receive funding.	- Generate knowledge; - Educate and create awareness among other actors; - Propose scientifically funded actions for e.g. regulation and procurement.	- Curiosity With - Reputation; - Funding;	- Institutional barriers to multidisciplinary and/or international cooperation; - Limited access to data and samples from industry.

When analysing how the actor types and their interests link to (possible) incentives and disincentives, we have proceeded from the most immediate source of pollution – the pharmaceutical industrial plant emitting pharmaceutical residue, in particular subcontracted producers of API. Below, we note some of the most important (dis) incentives for this category of actors and the relation between various producers (branded, generic, subcontracted) of antibiotics, as well as public actors in producer countries. We then focus on a crucial element of any viable solution, namely increased transparency in the supply chain. This for the simple reason that transparency is a crucial element for other actors to be able to act on pollution, since that requires information on who creates the pollution, to what extent, and what the supply chain looks like. After this, we examine the role of actors in consumer country states (Sweden), such as regulators and governmental institutions, but also pharmacies, media and hospitals.

Before considering possible options in some area in need of action, it is important to consider what actors are able to implement the suggested actions. How different actors are related to the area of industrial antibiotics is not always straightforward. The most obviously involved type of actor is the pharmaceutical industry (#1-#5 & #7). However, as we show in the Additional file 1, once the active pharmaceutical ingredients (or API’s) are produced by subcontracted companies (#3), these are often distributed across the globe by business networks (#1-#3, #7) which are complex and often opaque, at least for the final customers and users (#20, #23, #24, #26, #28). Through the mechanisms of global trade and linked regulation (#12) [20], the final antibiotic products eventually find their way to what we label ‘consumer countries’ (see Additional file 1 for more detail). Depending on local regulative systems (#14-#19, #21, #22, #30, #31) and market dynamics, different demands and prices are set in different places, and a combination of the commercial actors interact with each other, as well as further ones, in chains of distribution, retail and procurement. Although producer countries are also to some extent consumer countries, and many consumer countries are also to some extent producer countries (by hosting producers and distributors), we suggest that this distinction helps to understand the various interests in play.

India and China are the largest hosts of subcontracted producer of APIs (#3), at least for generic medicines. Europe and the USA on the other hand, host the largest research-based companies (#1), while also representing large and economically strong markets for the consumption of antibiotics. However, even though the majority of the final medicinal products available on, for instance, the Swedish market are produced in Europe, the APIs used by these European producers originate to a much larger extent from other parts of the world, often countries that have been ranked as those with in general ‘poor environmental standards´ [20, 24]. To simplify, consumer states often ‘outsource’ [10] the pharmaceutical pollution generated by their high consumption levels. For this reason, there will be differences between the typical interests of consumer states (#14), compared to producer states (#8), as the former have more indirect stakes in reducing emissions, while they have a direct interest in reducing health care costs.

Any of the types of actors described in the Additional file 1 can conceivably affect the extent of pollution by some type of action. At the same time, the actual choice regarding such actions will be guided by a wide variety of interests, and take place in a very complex landscape of other actors and interests. In what follows, we go on to examine in what way the actors may contribute to a solution and how the levels interact, analysing what incentives and disincentives may be created for producer actors, and for key actors in consumer countries, using Sweden and India as examples.

### The role of subcontractors in producing countries

Currently, there are limited legal or economic incentives for subcontracted producers (#3) to act in order to improve the situation regarding industrial antibiotics pollution. Recent initiatives by the pharmaceutical industry to address pollution indicate that the need to change is recognised primarily among some research-based companies (#1) [13, 25] but the economic costs involved disincentivize all producers from voluntary change – as evidenced by the fact that cases of substantial pollution are revealed regularly [10, 26]. Obvious costs of environmentally improved antibiotics production relate to more extensive efforts to avoid contamination of wastewater in the first place or to install effective treatment (#6). This may lead to a smaller market share, if the costs are reflected in increased prices, or otherwise result in a diminished profit margin. If the market shares remain the same while profit margin goes down due to increased production costs, this means that the value of the market is diminished, which in turn affects the future flow of new investor capital. Likewise, information on the level of pollution at individual production plants and how to assess these in a scientifically supported and reliable way is currently severely lacking [6, 10–12, 17, 27, 28]. For this reason, setting up reliable systems to monitor emission levels and types, and assessing when levels are acceptable and when they are not, would be a major research and development investment, involving substantial scientific, technical and staffing costs. The more that these costs have to be carried by individual production companies (#1-#3), the more they will constitute a serious disincentive of both these companies and their owners (#5), as well as states and individuals that benefit from their business (#8, #10), to go along with requests for better control of industrial antibiotics pollution.

One way to provide incentives for desirable change of subcontracted producers (#3) is, then, for other actors within the manufacturing country to take on (some of) the costs and the logistical arrangements needed for an effective management of industrial antibiotics pollution. For instance, the national government as well as regional governments (#8) may sponsor research and development, financially support technological arrangements, subsidize the cost of waste water treatment plants (#6) to assist in improved wastewater management, and so on. In addition, governments may offer softer incentives by initiating collaborative talks with industry and create benefit schemes for better performing companies. For example, the Chinese government stimulates their pharmaceutical sector and offers a competitive advantage by providing cheap electricity, land, water, and waste disposal to companies that perform well in various respects, including transparency measures and environmental control [29]. Finally, the government can, of course, add incentives through regulatory arrangements, laws, taxes, as well as charters for public agencies overseeing pharmacological industry, environmental safety, etcetera. Here, the national environmental agencies, such as the Central Pollution Control Board (CPCB) to take a specific Indian example (#9) may conceivably play a larger role in surveillance and enforcement of standards. However, to what extent the rather ambitious plans by Indian government to curb pollution will be successful is yet to be demonstrated [15, 30]. Note that all of these ideas shift the question to what incentives there are for these governmental actors to actually take up this role of incentivizing producers. The financial, logistical and infrastructural costs have to be paid somehow, at the end of the day by taxpayers who are also donors and voters (#10). For a politician – regional or national – this provides a disincentive to taking the action that would incentivize all of the other actors to pressure and assist the API producer.

Other actors that are in a position to incentivize subcontracted producers (#3) very directly would be their customers (contractors), i.e. research-based (#1) and generic drug producers (#2). If these actors start to require of their subcontracted producers to certify a good antibiotics pollution management, it would provide an immediate financial incentive for the latter to do so. That would also create an incentive for the state hosting the production (#8) to provide resources and institutional frameworks to support such a development. However, this raises the question how to incentivize research-based and generic producers themselves to exert such pressure.

One incentive for this chain of actors would be if there was an increase of internal national grass root political pressure, and here the (national and international) media, science and international organizations (#11, #13, #27, #29, #31- #33) may help to move things forward by creating awareness of the potential risks associated with industrial antibiotics pollution. However, such change usually moves slowly, and the question then arises what can be done to incentivize action in the meantime. In addition, while awareness may certainly move political opinion, it is far from certain that it would suffice to balance the mentioned disincentives, in particular the financial, logistical and infrastructural costs.

### Transparency in the supply chain

One important disincentive for action is the current general opacity of the production and supply chain – for example simple information on what company produces the API in a given product and where that production takes place is considered confidential and is usually only available to the authorizing agencies to facilitate quality control. This makes it a complicated task for governments and institutions (#8, #14- #19, #30, #31) to create effective systems for pollution control, or for third parties (including consumers #28 and media #32) to exert pressure by linking sometimes apparently polluting production sites abroad to the companies selling the final products in consumer states [18, 20]. Pharmaceutical companies may, of course, be motivated by reputation concerns to avoid being perceived as antibiotics polluters. Rankings of public perception [31] may have a significant impact on certain companies’ behaviour, but primarily those that consider branding a critical issue of their business (#1). Again, this indicates that there is an important role for scientists and media in revealing cases of substantial pollution. Environmental agencies and NGO’s, both local and global, may play a similar role in appealing to companies´ reputation concerns. NGO’s may not only apply political pressure, but can also contribute by gathering data and documenting pollution and its effects. By way exerting political pressure, such strategies can enable for example patients, hospitals, pharmacies and prescribing physicians (#20, #23, #24, #26, #28) to exercise their power to apply pressure – we will further expand on how they may do this below.

Reputation concerns are particularly important for research-based companies (#1), as these have a brand to protect in the eyes of public perception. In contrast, generic producers (#2) and subcontracted producers (#3) do not, or at least not to the same extent, have a need to protect reputation in order to stay in business, as their business model largely builds on providing quality products to a low cost. Reputation may also indirectly concern many large investors in pharmaceutical companies (#5), regardless of whether the companies are research-based or generic producers. Financial institutions, such as banks, insurance companies and pension funds, have been shown to be sensitive to pressure from consumers and advocacy groups (albeit slowly and not always effectively, see [32, 33]). However, since protecting the reputation of a market brand is connected to whether a company *seems* to act in a responsible and sustainable manner, not just whether they actually are, there is a lack of incentive to push for a more transparent system. Companies can be reluctant to share information to make the production chain more transparent for the very reason of protecting their reputation. These disincentives will also likely affect investors. Paradoxically, therefore, reputation concerns may provide *disincentive*s to effective antibiotics pollution action. While pharmaceutical companies show an increased willingness to take antibiotics pollution seriously, [13] given the current lack of transparency of how industrial antibiotic emissions link to specific producers, very few are willing to disclose information that would make the situation less opaque, such as identifying who their subcontractors are and what levels of antibiotics they emit to the environment [14]. Authorising agencies, such as the LV (#15) and the EMA (#31) do dispose over this type of information, in order to ensure inspections aimed at quality control, but they are not allowed to publicly disclose this information [18, 20].

However, *had* the production and supply chains been reasonably transparent, the incentive coming out of a research-based company’s concern for reputation would seem to have pulled in the opposite direction. Once a company is tainted by a polluting production chain, it starts to make good sense for research-based producers (#1) and their owners (#5) to avoid being associated with such chains and promote those with a higher level of sustainability. The higher the awareness and concern about industrial antibiotics pollution is, the stronger the rationale is not to be associated with it. This is for example the case in Sweden, and increasingly so in other high-income countries. Based on this, research-based companies acquire reasons to pressure subcontracted producers (#3), and thereby initiate a chain of incentives for constructive change supported by producer countries (#8) in line with what was envisioned above. However, generic producers (#2), as observed, will not be incentivized to the same extent (as their brand is not as critical to protect), which means that insofar as the sales for generics are not that sensitive to improvement in transparency, these producers will be less incentivized to pressure subcontracted producers. Unfortunately, this factor will then add to the disincentives of research-based companies to work for increased transparency of the production chain, since this would put them at a relative business disadvantage when compared to the generic producers. While generic producers would be able to buy APIs more cheaply from subcontractors, research-based companies would have to carry parts of the costs for a more sustainable API production.

It should be clear at this point that understanding how markets function is important for the analysis of how to incentivize producers, and relevant actors in producer countries, towards more sustainable practices with regard to industrial antibiotic pollution. In a market, the *relative* advantages and disadvantages of stakeholders are in the forefront of how business actors (and those with interests closely linked to these) assess the attractiveness of a more environmentally sustainable strategy. Unless there is an added benefit, companies are understandably reluctant to exert themselves overly when the competition is unlikely to incur similar costs. This strategic consideration illustrates the importance of finding ways to incentivize all types of producers (#1-#3) equally, in turn pointing to the importance of creating internationally harmonized policies and to an important role for internationally coordinating institutional actors (#12, #13, #30, #31). On the other hand, there is a danger in strategies that rely on all relevant actors being on board: aiming for global or multinational consensus across many or all actor types may well lead to a situation where actors passively await action by others.

### The role of actors in consumer countries (Sweden)

Given the multinational and complex nature of both the drug market and international politics and trade, the relevant actors involved are not confined to those within the borders of the countries where the production – and therefore the pollution – takes place. As outlined in the Additional file 1, the production of API’s cannot be seen in separation of the international supply chains and the background of the international regulative landscape. This observation moves our search light from the producers and producer countries to those that dominate consumption.

Although, as we will show, there is good reason to look at consumer countries in developing a response to industrial antibiotic pollution, there are significant obstacles in the way. A disincentive that stands out is an unwillingness to accept increased healthcare costs, as all of the incentives for industry mentioned can be expected to be reflected in pharmaceutical prices. Systems for generic substitution of drugs that we will discuss below are a particularly institutionalized example of this disincentive. However, there are various ways to counter such obstacles. Below, we highlight licensing, reimbursement and procurement mechanisms. It should be noted that media and scientific researchers (#32, #33) play a role in increasing awareness among institutional and political actors in high-income consumer states such as Sweden (#14 - #25), and international political collaborative organisations, such as the EU and the UN (#12, #13, #30, #31), help to promote this sort of development. We will start by mapping out potentially effective ways to create incentives for actions that may bring about positive change, and after this list disincentives to such actions actions.

One powerful tool to motivate pharmaceutical companies is through licensing requirements and the routines of institutions in charge of implementing these (#15, #31). In the EU, licensing of medicinal products is to a large extent harmonized, which offers promising opportunities. When applying for a European marketing license, pharmaceutical companies are required to submit an environmental risk assessment (ERA) to the EMA or a national competent authority within an EU country [34]. The ERA covers a broad set of environmental assessments with a clear possibility to incorporate considerations relating to antibiotic resistance. *However, to date, such risks are not covered within this regulation*. Also, only pollution associated with usage (not production) is covered within the ERA. The need for amending the ERA to bridge these two gaps have been pointed out earlier [17]. At the same time, in practice, there is no penalty for non-compliance and products are not withheld from the European market based on non-compliance with the ERA [17]. In contrast, the *risk-management plan*, which assesses amongst other things the safety of the medicine for the patient, is used to determine eligibility for a license on the European market, and similar mechanisms may be used by national agencies. Addition of an amended ERA to cover industrial pollution as a required ingredient in the risk-management plan (and equivalent national requirements), would therefore produce a substantial incentive for companies to mind about the sustainability of their production chain. In addition, it would be a basis for licensing agencies to require transparency of this chain for the purpose of assessing the environmental footprint of a drug, specifically with regard to antibiotic pollution. Of course, in order to accurately offer such assessments, in turn, it would be important that such a move is accompanied by research on the risks of industrial pollution. We return to this point later. Such a requirement may be applied to both research-based and generic producers, and may be extended also to parallel import distributors in regulations for permission to trade in drugs, all in order to create equal conditions on the market. At the same time, the nature and function of the risk assessment involved in the licensing of drugs means that considerations relating to pollution would have to be weighed against other considerations, such as need, efficacy, safety, and the availability of alternative treatment [17]. This means not only an uncertain outcome, but also possible risks of ethical controversy and political backlash [35].

More robust incentives of this kind could be created through the fact that pharmaceutical companies active on the European market are bound by the EMA *Good Manufacturing Practices* [36]. These are not relative in the way that single risk factors in the risk management plan are. Although currently environmental considerations are not considered in the GMP, they could be devised to include discharge limits, requirements of disclosure with regard to production chains, and other relevant demands to secure a minimum level of sustainable production practices. As the possibilities to follow up on requirements and effectively control compliance are currently limited [10, 17], such control would require stronger international collaboration, something on which the European Commission hints in the ‘EU action plan’ [37]. Unfortunately, however, the action plan remains vague about the specifics of such support, and focuses mostly on the value of more knowledge, without committing to robust incentives in the pharmaceutical market, let alone licensing practices. To get the ball rolling towards incentivizing research-based and generic producers to become more transparent with regard to production chains, and to require of subcontracted API-producers to monitor and disclose emissions, as well as to mitigate these emissions to acceptable levels, explicit regulatory requirements seem to be necessary. This probably requires national and international political action by high-income consumer states (#12-#14, #30), but once that ball is set in motion, incentives for institutional actors to assist and press industry actors and producer country institutions will likely follow. What could be such a starting point are the discharge limits for 111 antibiotics we proposed in late 2015 [26]. These were immediately highlighted in the British AMR review [38] and later adopted as voluntary target concentrations by many leading antibiotic manufacturers [13] and they have also become the basis for the proposed Indian regulation [15].

As mentioned, licensing agencies have access to data on the origin of API’s, but they are not allowed to publicly disclose this. A complementary action is therefore to require that information detailing the production sites of antibiotic products are made publicly available, as this would open up the environmental performance of the production chains for public scrutiny. Such information could be given on websites, (for example the producer’s own or that of the European Medicines Agency (#31)) alternatively labelling on containers (which is highly regulated) or pharmacy shelves [39]."(see below)" The information could include the exact location of where the constituting API’s are produced and formulated (a mention of only the country or region of origin would have very limited value [20]), the companies involved in these stages of the drug production, as well as information on applied discharge limits (voluntary or enforced) and the documented level emission control. However, this type of strategy becomes effective only when it feeds into a consistent customer demand mechanism that will create incentives for companies to offer products produced under demonstrably sustainable conditions. When the distribution of drugs in general, and antibiotics in particular, is controlled directly or indirectly (for example through prescription guidelines) by national or regional institutional actors within consumer countries, this may be the case to the extent that these actors make explicit requirements with regard to production chain disclosure and guaranteed emission levels. However in large parts of the world, particularly in low- and middle-income countries, the market in antibiotics is not consistently institutionalized and there are no, or very weak, prescription requirements, and most health care and pharmacy is operated through small-scale private actors [40]. In these health systems, increased public awareness created through actions by the media, NGO’s, patient, professional and academic actors (#27, #29, #32, #33) may help to produce some effect. However, in both cases the effects of such incentives are uncertain. Once again, this moves the search light to consumer countries, and the way their internal actors choose to position themselves. For those countries that lack institutional control over drug distribution and consumption, the first step would then be to create the needed frameworks, admittedly a formidable political task for these consumer states. In the following, we limit ourselves to high-income consumer countries that do have such frameworks, taking our specific examples from Sweden.

The Swedish government (#14) has so far addressed the issue of the environmental dimensions of pharmaceuticals with greater force than the EU (#30), although there is some recent progress in the European context as well [41]. For example, on instruction from the government, the licensing authority *Läkemedelsverket*, LV (#15), has explicitly addressed the issue of pharmaceutical pollution [42]. However, the resulting incentives are still limited, due to similar factors as already mentioned in the case of EMA. Although LV has made constructive proposals to incorporate more stringent demands in ERA and Good Manufacturing Practices as formulated by the EMA, they have little, if any, room in this respect to pursue a course independent from the European context. A revised GMP framework would put equal demands on all antibiotics sold on the European market, but progress in this direction has been slow.

Additionally, a Swedish government white paper [43] has suggested that an alternative way of creating incentives for environmental adaptation lies within the framework of the pharmaceutical reimbursement system, covering part of the costs for prescription medicines [43]. During the reoccurring process of assigning which single product (out of often several clinically interchangeable products) that should be subsidised, the idea is that an environmental premium should be given to those products/manufacturers that meet certain criteria (yet to be defined) relating to emission control, thereby making it easier for them to be selected, despite that they may not offer the least expensive product. It is clear that such a system, if the premium is sufficiently high, may do much to address the issue of stimulating greater transparency and offer a strong concrete business incentive for industry to curb emissions. An immediate question that arises, however, is how such a system should be designed, who would end up paying the cost, and how motivated key political actors will be to accept them. In addition, a well-functioning premium system involves calculating the environmental costs, establishing criteria for conformity etcetera [43].

The LV also makes decisions on the interchangeability of drugs within the Swedish ‘generic substitution system’ [44]. The Swedish system dictates that if two drugs are interchangeable, the cheaper of the two will be given preference in the subsidizing and healthcare procurement system. The criteria for interchangeability relate solely to clinical risks and benefits, meaning that generic producers with lower production costs are often favoured against research-based producers and there is no special consideration of environmental considerations. However, given these decisions on interchangeability, the public subsidy authority Tandvårds- och läkemedelsförmånsverket (TLV, #16) may act within its authority on the allocation of the public subsidy of drugs. When comparing products which are considered interchangeable, the TLV could theoretically include pollution criteria, such as requirements related to industrial pollution [43]. This would incentivize not only research-based producers, but also generic producers. Yet, given the current assignment by the government instruction, there are no such pollution criteria and TLV has no authority to place them. Thus, TLV appears to have very little room to make substantial environmental demands. Furthermore, in the EU (and Sweden), this particular incentive will be limited, as generic substitution only comes into play after a patent has expired [43, 45]. On the other hand, the overwhelming amount of antibiotics (in terms of doses, not necessarily money) are for antibiotics whose patents are indeed expired [20].

Finally, either the Swedish government, or the EU collectively, may attempt to exert influence by levying taxes on products that do not fulfil certain environmental criteria. Although it is beyond our expertise to comment on the legal opportunities for such a solution, we acknowledge the challenges involved in agreeing on criteria and determining an effective, still acceptable, taxation level, especially in an opaque context. These problems are further exacerbated by the complicated task of reaching agreement on taxes and the ways to implement them, especially in an international context.

All of the governmental actors mentioned above may thus act to include in their decision rationales considerations related to industrial pharmaceutical pollution, although not in all cases under the current statutes. This may take the form of both an internalized external cost that counts as a reason against buying or prioritizing a drug, and of a benefit when companies demonstrably act to make pollution management more sustainable. However, as documentation is lacking on the basis of which the necessary assessments for such policies could be made, more information regarding the actual level of pollution management would be required [11, 43].

### The role of other stakeholders in consumer countries

In theory, pharmacies could also be an important actor with regard to generic substitution, having patients select the environmentally more preferable among therapeutically equivalent drugs. In Sweden, however, they would be constrained by the generic substitution system, and the decisions by LV and TLV for prescription drugs (which all antibiotics are). Unless these latter actors weigh environmental factors into their decisions, this remains impossible for pharmacies. In states where pharmacies have a license to act more autonomously, this role may remain open. Another way in which pharmacies may contribute to the right incentives, is by labelling practices. For example, there is in Sweden a voluntary labelling initiative by the private pharmacy chain Apoteket Hjärtat, which involves labelling on the shelf. This labelling is, however, limited to procedural company characteristics, whether they follow a certain reporting system and are part of the “pharmaceutical supply chain initiative” [39, 46]. Also, and more importantly, such labelling practises by the pharmacies are not applicable to prescription drugs.

Separate from the pharmacies (and thus the issue of interchangeability), incentives may be offered in the context of public procurement [47, 48]. As described in the Additional file 1, Swedish public hospitals and primary care clinics (#20) are usually either owned or subcontracted by the county councils (#21), who buy and negotiate antibiotics. The amount is relatively minor when compared to the total amount of the antibiotics that are used in Sweden, but still substantial. In addition, county councils subcontract to privately owned hospitals and clinics. County councils are therefore in a unique position to lay down environmental demands, and therefore may contribute an important piece of the puzzle in providing effective incentives to pharmaceutical companies. Nationally, the public procurement agency, Upphandlingsmyndigheten (#19), in turn, can help guide county councils in a supporting advisory role. In addition, the inter-regional body for national price negotiation and priority setting of new treatments within the organization of counties and municipalities, especially NT-rådet (#22) [49] can help counties act jointly and effectively by taking environmental considerations into account when assessing the overall value of a drug, and make environmental demands in these negotiations. Additionally, county government (#21), which has the final mandate to decide what treatments are “on the menu” to be procured by public hospitals and primary care, may take similar steps with a similar impact.

Another possible pathway to create incentives based on LV and EMA decisions on drug licenses is clinical guidelines. We have already mentioned regional committees issuing recommendations on what drugs to use for given indications (#21). At the same time the national agency, the Socialstyrelsen (SoS) (#17), has a powerful and coordinating position role in issuing national treatment guidelines. Given the strategic threat to the quality of healthcare generally, it may not be unreasonable to have such guidelines allow room for environmental health considerations as part of the rationale. The government (#14) may give SoS such a charter, and direct it to collaborate with the Public Health Agency of Sweden (FHM) (#18) in this endeavour.

All ideas of changing the system for drug substitution conflict with the underlying policy rationale of this system. The point of such systems is to reduce the public cost for pharmaceuticals by exploiting the competition between research-based and generic producers. Generic drugs ruled to be interchangeable are given fast access to the market and public subsidy, thereby making cheaper alternatives quickly procurable for healthcare. Measures as the ones mentioned above mean that generic manufacturers (which are not as sensitive to other pressures to control environmental emissions) will probably lose competitive advantage. At the same time, there is a strong political interest in reducing healthcare costs, linking to an interest across large segments of the public in avoiding tax increases. This creates a disincentive against implementing reform of the drug substitution system that could incentivize producers to better control industrial antibiotics pollution. This disincentive is an unusually salient and specific example of how widely embraced political aims to reduce pharmaceutical cost in publicly funded health systems may undermine motivation to take action that could incentivize industry and producer countries to control industrial antibiotics pollution. Simply, put, whatever actions are taken, these will probably be reflected in drug pricing, and thus run contrary to currently prevailing economic health policy aims on of many high-income consumer states.

In the long run, of course, this disincentive weighs quite lightly compared to the primary value of having an effective health system, which is in turn threatened by antibiotic resistance. However, longer time horizons are seldom very decisive in politics, unless there is a stark public awareness of very serious threats. Here, as before, media (#32), NGO’s (#11) and academic and professional actors (#26, #33) may act to shape a public atmosphere weakening this disincentive. Especially academics and health care professionals are in a key position to provide the understanding and information in order to prompt such a development, since, although typically their audience is limited, scientists and health care professionals play a crucial role in making the relevant facts public.

A recurring disincentive for many of the actions that could be taken by national institutional actors has its root in well-known collective action problems related to global problems [50, 51]. There are Swedish county councils (#21) that have requested from pharmaceutical companies that emissions are monitored during manufacturing, but on their own their negotiating power vis-à-vis multinational corporations is weak. Actors within the counties, such as single hospitals (#20), the Läkemedelskommitté (#21), or pharmacies (#24) have even less power in this respect. For this reason, nationally coordinating institutions like LV (#15), TLV (#16) and NT-rådet (#22) are better able to act in this respect, but we have also seen that their mandate is limited. Therefore, the counties here primarily face an incentive to start acting even more in coordination, in order to create an incentive for each to use the procurement and priority setting incentive. However, even if the mandate would be expanded by the state (#14) to facilitate also more effective and well-coordinated national action, the state level actors face a situation where the difference that they can make on their own is limited. Sweden has a relatively small mass in terms of drug industry revenue (0.9% of the global market value of pharmaceuticals), suggesting that any effective intervention of the types mentioned is likely to be rather costly. If several countries together, the EU (#30), or better yet the global community, were to formulate joint demands, this would result in a much more powerful position to incentivize large international industrial conglomerates. However, as we have already seen, in the European context, let alone the global one, the legal power of the relevant institutions is relatively weak: the EMA (#31), for example, has as its primary purpose streamlining the national policies decided by single states, not initiating effective joint action. This is a disincentive against EMA acting in the direction sketched earlier to create incentives for pharmacological companies to mind more about environmental factors. And as we move into global institutions (#12, #13), while potential bargain power becomes much stronger when these do succeed to act forcefully, the political base to further an independent agenda to restrict the policy options of sovereign states proportionally weakens. Therefore, at the end of the day, this disincentive can be mitigated or removed only to the extent that a sufficient number of single high-income consumer states are motivated to coordinate effective action to incentivize producer states and industry. Once again, academic and professional actors, NGO’s and the media have a key role in incentivizing such motivation.

Another underlying disincentive to effective institutional action (on any level) is the current lack of effective surveillance systems and systematic emission data on industrial antibiotic pollution [10]. This is the case because effective action requires specific information to act upon, but also because such information affects prioritization in order to direct incentives to the appropriate targets. This holds on the national (Swedish or other single consumer states) as well as the multinational (EU and, ultimately, UN agencies) level. Therefore, a first step needs to be to incentivize better *industry transparency* with regard to production chains, as well as *demonstrable actions to monitor and control emissions* at the source [18, 20]. Lack of transparency and documented action in this respect creates an environmental health uncertainty that can be viewed as an external cost for society that needs to be internalized in different ways by different actors, thereby making a drug less attractive for license, clinical recommendation, subsidy, procurement and finally use. The higher this cost is estimated by a societal institutional actor, the more forceful the incentive for companies to act in order to favour their own products. Once a system of reasonable transparency regarding production chains and quality assured monitoring of emission levels is in place, the disincentive for societal actors is gone and the search light can move to actual emissions, so that companies can be rewarded for assuring effective curbing of antibiotics pollution through measures at their own plants, or pressuring on subcontracted partners.

## Conclusions

**Fig. 1:**
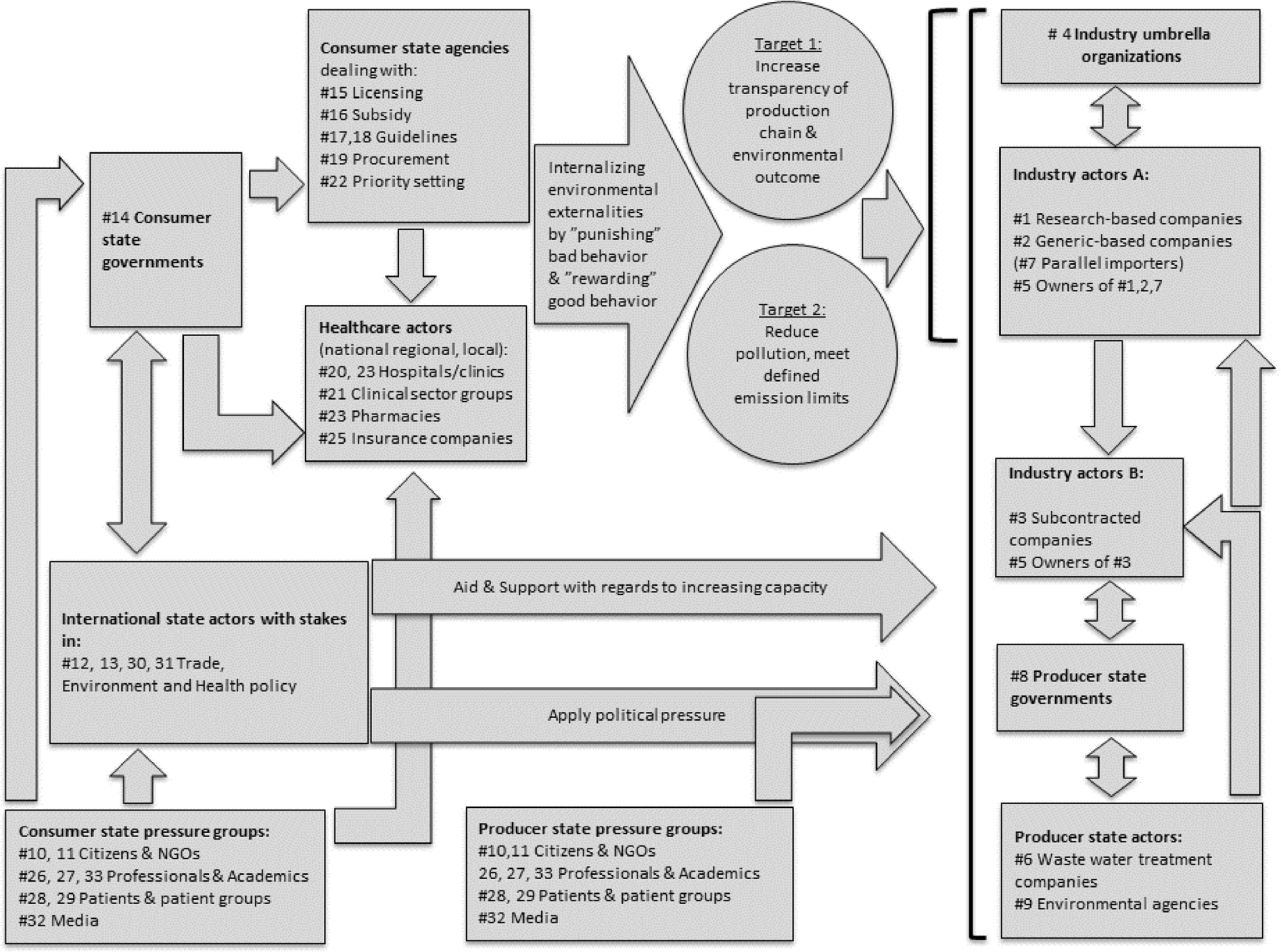
Examples of chains of actions of different actor types to incentivize action of other actors to improve the management of environmental industrial antibiotics pollution

In this paper we have mapped important incentives and disincentives with regard to possible ways in which actors that are directly or indirectly involved in the issue of industrial antibiotic pollution can be motivated to effective action (incentives), as well as to obstacles that would hinder such action (disincentives). Taking account of all of the actor types, and their possible incentives and disincentives to act effectively in this area, as described in the *Results* section, we may plot typical chains of actions and resulting incentives (including overcoming noted disincentives), and use this as a starting point for actors to decide how to design policies and specific measures and where, more exactly, to direct these. Some examples of such incentive chains, starting with public actors in consumer countries, and ending up with better pollution management by API producers, are illustrated in Fig. 1.

The collective action challenge presented by industrial antibiotic pollution is an outcome of how effective incentives to combat industrial antibiotics emissions rely on action on different levels and how these levels interact in sometimes rather complex ways.

We have presented a first step to a social network analysis in order to map these various actors and their interactions. This interconnectedness, and the complexity it involves, is itself a feature of the problem, and needs to be taken into account when considering solutions. We have shown how incentives for actions to curb industrial antibiotics pollution are unlikely to arise without considerable support action by consumer countries and the international community. We have also demonstrated that the creation of systems to make production chains transparent, and emission levels controllable, plays a crucial role in addressing the problem of antibiotics pollution. Without such systems, very little actions of any other kind seem likely to bring about positive change, no matter what actor perspective we take.

The combination of these three challenges (complexity, international dependency and need for better transparency) are common to various ethical problems of a systemic and global nature: think of diverse global topics, such as climate change, human rights issues in the production of globally traded consumer goods, animal welfare in meat production and the use of pesticides in agriculture. For each of these areas, a relatively large number of different actors constitutes a complex chain or network in which each of them is dependent on what the others do.

As noted, some possible actions may create tensions between different normative considerations. For instance, incentivizing through reform of drug licensing and clinical guidelines may create provocative tensions between long-term environmental health considerations related to industrial antibiotics pollution and ordinary short-term clinical ethical considerations regarding benefit and safety for patients in care. As mentioned, political normative conflicts between short-term aims to reduce healthcare cost may also conflict with many incentives that can be created by consumer state actors. We do not argue for any particular resolution to such dilemmas, neither have we come to any specific conclusion on what from a normative perspective is the right course of action for the various actors involved, although we have pointed out some normative considerations. This brings us to the delicate question of responsibility.

A knee-jerk response to the problem of pollution may be to simply blame the ones who pollute. While we by no means want to absolve those who discharge antibiotics, we think it should be clear however that in an issue as diffuse as this, there is no single individual actor that can be blamed as the sole culprit. And even if we could – this would still not free us of the obligation to think about possible solutions, given the fact that some actors fail to do as they should. Our suggestion here is that, when thinking about solutions in that light, there is a special role for governments of consumer countries. We have three reasons for this.

First, producer country states will not be able to solve the issue on their own. Although the opportunities to effectively and rapidly change legislation with regards to emission are larger from within each producer country, environmental regulatory measures alone will likely not suffice. This means that we should also look at economic incentives and aid for capacity building in producer countries. Those actors that have more financial resources to dispose over, will be more likely to be able to change the dynamic described in this paper. The mechanism here is simple: the buyers of products have influence to the extent that they represent more economic mass; the big buyers of pharmaceutical products (the EU and the USA) thus represent more economic power, especially if they coordinate their actions.

Second, we have shown that there is a central role for information when we look for possible solutions to create better transparency. Once the information is available, effectively aligned systems in consumer countries may stimulate sustainable production models. Transparency and controllability are thus crucial to the success of any attempt to address systemic and global issues, and this for two reasons. On the one hand, transparency and controllability provide quality assured insight into the problem and how possible solutions may proceed. On the other, the notion of international cooperation requires that actors have knowledge of what the others are doing and how joint action pays off. This is not necessarily an indication of mistrust, but a condition for creating meaningful options for action that can be viewed as legitimate from the different perspectives of different types of actors and different states. In order to address complex issues such as industrial antibiotic pollution, we need to consider in what ways actors can create space for other actors to acquire information that is important for them to act effectively downstream. Viewed in this way, pursuing the construction of systems and standards for transparency and control will likely mean very different things on different levels. However, we have seen that there are good opportunities from the legislative perspective of consumer countries to affect the transparency in the production and supply chain. Specifically, we have seen that licensing, adapted reimbursement systems and procurement may offer ways to enhance transparency.

Third, there is a need for increased knowledge on for example the extent of emissions, the effects of pollution on the emergence of resistance in pathogens, as well as the effectiveness of various economic pressure mechanisms. Hence, a multidisciplinary approach is needed to bring science all the way to policy. We need to stress that there is sufficient consensus on the risks involved already to initiate measures, but better knowledge is likely to facilitate more effective actions. Research initiatives should be largely funded from within high-income consumer states, for the pragmatic reason that they have more financial resources. Yet collaboration on a global scale is vital to success. Communication of existing knowledge, creating awareness among stakeholders, is also key.

These three observations point strongly to the conclusion that the greatest opportunity to create incentives are mostly located in consumer countries, preferably in collaborative concert. Given the complexity of the network, the room to manoeuvre can be quite limited for many actors. Less so, however, for some of the actors in consumer states, and in particular not for those on the level of consumer state governance. We argue that here is a strong duty for actors who can do something about the problem to actually take action. Exactly how the most effective mix of regulation, aid and “carrots and sticks” should be designed will likely vary depending on the economic, societal, legal and cultural context. The analysis presented in this paper suggests several explicit actions where a chain of incentives and disincentives may be influenced in order to effect change.

## Supplementary information

**Additional file 1.** Relevant actor types and their interests.

## Data Availability

Data sharing is not applicable to this article as no datasets were generated or analysed during the current study.
