# Correction to: Managing pollution from antibiotics manufacturing: charting actors, incentives and disincentives

**DOI:** 10.1186/s12940-019-0545-8

**Published:** 2019-12-12

**Authors:** Niels Nijsingh, Christian Munthe, D. G. Joakim Larsson

**Affiliations:** 1 Centre for Antibiotic Resistance Research (CARe), at University of Gothenburg, Gothenburg, Sweden; 2 Department of Philosophy, Linguistics and Theory of Science, University of Gothenburg, Gothenburg, Sweden; 3 Institute of Ethics, History and Theory of Medicine, Ludwig Maximilian University, Munich, Germany; 4 Department of Infectious Diseases, Institute of Biomedicine, The Sahlgrenska Academy, University of Gothenburg, Gothenburg, Sweden

**Correction to: Environ Health (2019) 18:95**

**https://doi.org/10.1186/s12940-019-0531-1**

Following publication of the original article [1], the author explained that there are multiple errors in the original article;

1. Incorrect Additional file 1 was uploaded and is replaced with the correct Additional file 1.

2. Several references (52–70) were incorrectly placed in the references list and they are now have been removed and placed in the supplemental material.

3. The format of Table 1 was incorrect formatted and this is now corrected.

**Table 1:** Thirty-three actor types with possibilities to contribute to the reduction of antibiotic emissions from manufacturing. For each actor type, examples of their interests, possible actions and incentives and disincentives for those actions are listed (more details to be found in the main text and the supplementary material). Broadly common interests among actors, e.g. individual’s/employees desire to contribute to positive societal change (reducing pollution, improving public health) are implicit but have not been listed for each actor. For actors #15,16,17, 21 and 22 we have listed Swedish examples of actors types

Actors	Interests	Actions	Incentives	Disincentives
# 1 Research-based pharmaceutical companies	- Increase turnover, reduce costs; - Strategic interests (e.g. “stay ahead of the curve); - Reputation concerns; - Preserve effectiveness of product by curbing antibiotic resistance.	-Motivate #3: demand good pollution control for the API’s they buy; - Monitor & reduce their own discharges; - Set internal discharge limits; - Act transparently with regards to production sites (also of suppliers) and environmental performance.	- Emission standards; - Legal requirements; - Economic incentives (price, costs, turnover); - Pressure from investors and buyers; - Reputation concerns.	- Transparency as a threat for reputation concerns; - Higher production cost; - Lack of follow-up of external demands - risks of unfair competition.
#2 Generic pharmaceutical companies	- Increase turnover, reduce costs; - Strategic interests (e.g. “stay ahead of the curve”); - Preserve effectiveness of product by curbing antibiotic resistance.	-Motivate #3: demand good pollution control for the API’s they buy; - Monitor & reduce their own discharges; - Set internal discharge limits; - Act transparently with regards to production sites (also of suppliers) and environmental performance.	- Emission standards; - Legal requirements; - Economic incentives (price, costs, turnover); - Pressure from investors and buyers.	- Higher production cost; - Lack of follow-up of external demands - risks of unfair competition;
# 3 Subcontracting pharmaceutical companies	- Increase turnover, reduce costs; - Strategic interests (e.g. “stay ahead of the curve”); - Preserve effectiveness of product by curbing antibiotic resistance.	- Monitor and reduce their own discharges; - Set internal discharge limits; - Act transparently with regards to environmental performance.	- Emission standards; - Legal requirements; - Economic incentives (price, costs, turnover); - Pressure from investors and buyers (i.e. #1 & #2).	- Higher production cost; - Lack of follow-up of external demands - risks of unfair competition;
# 4 Umbrella organisations/ collaborations between pharmaceutical companies.	- Represent members (#1, #2, #3); - Align interests of members.	-Coordinate action	- In addition to those applying to #1,2 and 3: become a stronger force for promoting common interests.	- Interest and priorities may differ between members.
#5 Owners of pharmaceutical companies	- Profit on investment; - Reputation concerns.	- (Threaten to) withdraw investments in #1, #2 and #3; - Power through representation in boards.	- Pressure from customers and interest groups; - Financial incentives (risk for loss of business associated with “scandals”).	- Limits on (short-term) profits, as owners set profit expectations.
# 6 Waste water treatment plants (WWTPs)	- Increase turnover, reduce costs.	- Implement more effective treatment; - Monitor and report emissions.	- Government legislation; - Subsidies.	- Costs.
# 7 Parallel importers	- Increase turnover, reduce costs.	- Promote transparency and regulations.	- Pressure from buyers; - Legislation.	- Very limited ability to gain information on, or to influence the production chain
# 8 Producing country states	- Represent population; - Protect public health; - Protect economic interests.	-Regulate industry in terms of emissions; -Pressure, negotiate with #1–3; - Sponsor research and knowledge transfer; - Support infrastructure.	- Political pressure from citizens and interest groups; - Treaties, multilateral agreements, foreign pressure; - Public health concerns.	- Economic interests: protecting current industry - strict standards may create disadvantages for national producers; - Lobbying by #1–3.
# 9 Environmental oversight agencies	- Follow statutes and directives as defined by #8; - Protect the environment (and public health)	- Implement and enforce rules and regulations; -Provide data on emissions.	- Pressure from various actors; - Directives deriving from #8.	- Pressure from #1–3, in particular on the local level.
# 10 Citizens of producer states	- Economic concerns; - Public health;- environmental protection.	-Pressure industry and government; -Vote.	- Awareness; - Economic, health and environmental interests.	- Lack of information/ awareness; - Lack of interest; - Lack of effective political power.
# 11 Citizen interest groups, environmental and human rights NGOs.	- Represent #10; - Represent particular interests.	- Coordinate action; - Create awareness; - Exert pressure.	- Pressure from supporters; - ‘Mediagenic’ action may be more attractive with an eye on public support.	- Limited political power.
# 12 Inter-governmental political forums (eg. G7)	- Coordinate and represent national and international interests.	- Apply political pressure; - Harmonize policies.	- Input by goverments, political leaders; - Pressure by interest groups, political organisations etc.	- Many different interests, they may not always align.
# 13 United Nations agencies	- Initiate and harmonize collective action on global problems.	- Create awareness; - Harmonize policies across nations; - Exert pressure on industry and governments.	- Pressure from governments, interest groups, political organisations etc.	- Limited power.
#14 Consumer country states	- Represent population; - Protect public health; - Protect economic interests.	- Regulate; - Establish premiums; - Direct research funding; - Direct actions by national agencies; -Influence other consumer states and # 30.	- Political pressure by citizens and interest groups; - Treaties, multilateral agreements, foreign pressure; - (Global) public health. Concerns.	- Economic interests: costs - Lobbying by #1–3; - Little mass, individually (higher cost to establishing premiums); - Institutional barriers (eg. state generic substitution system).
# 15 National Licensing agencies (*Läkemedelsverket*, LV)	- Follow statutes and directives as defined by #14; - Good, affordable health care.	- Implement standards and licensing of medical products;	- Steering by national government.	- Limited mandate.
# 16 Agencies committed to subsidizing decisions (*Tand- och läkemedelsfömånsverket*, TLV)	- Follow statutes and directives as defined by #14; - Good, affordable health care. - Effective resource allocation.	Potentially (but not currently): - Weigh environmental concerns in reimbursement decisions.	- Steering by national government.	- Limited mandate; - Limited possibilities for action under current statutes.
# 17 Agencies committed to prescription policies (*Socialstyrelsen*, SoS, and *Inspektionen för vård och omsorg*, IVO	- Follow statutes and directives as defined by #14; - Good, affordable health care.	- Issue national treatment guidelines (in cooperation with # 18).	- Steering by national government.	- Limited mandate.
#18 Public health agencies	- Follow statutes and directives as defined by #14; - Good, affordable health care.	- Issue national treatment guidelines (in cooperation with # 17)	- Steering by national government.	- Limited mandate.
#19 Agencies committed to public procurement: *Upphandlingsmyndigheten*	- Follow statutes and directives as defined by #14; - Good, affordable health care.	- Supporting #20, 21 and 22 to put pressure on # 1 and 2	- Steering by national government.	- Limited power.
#20 Public hospitals and clinics	- Follow statutes and directives as defined by #14 and #21; - Represent interests of #26 & #28; - Effective resource allocation.	- Apply environmental criteria in procurement; - Improve awareness.	- Regulation.	- Pressure on cost-efficiency; - Limited negotiating power.
#21 Regional government (county council) and their regional medical products committees (*Läkemedels-kommittér*)	- Represent population; - Protect public health; - Good, affordable health care; - Protect economic interests.	- Steer #20; - Weigh in environmental concerns in regional treatment recommendations.	- Political pressure by citizens and interest groups; - National policies; - Public health concerns.	- Limited power.
#22 Central priority setting organisation for drug procurement (*NT-rådet* & *Sveriges kommuner och landsting*, SKL)	- Effective resource allocation; - Good, affordable health care.	- Help counties act jointly and effectively.	- Steering by national government.	- Limited mandate.
#23 Privately funded and operated clinics and hospitals	- Profit; - Promote and protect health of their patients.	-Apply environmental criteria when buying antibiotics.	- Demands made by subcontracting county councils; - Pressure from #28, 29.	- Very little negotiating power.
#24 Pharmacies	- Profit; - Reputation concerns.	- Take environmental concerns into account when purchasing antibiotics (applicable to some countries, not all); - Improve awareness.	- Media attention; - Attracting costumers.	-Little or no influence over what antibiotics to provide through governmental restrictions (in some countries, but not all).
#25 Insurance companies	- Profit; - Reputation concerns.	- Negotiate, pressure # 1,2.	- Financial considerations (e. g. premiums, or taxes).	- Interest in lower prices.
#26 Physicians and other health care professionals	- Economic interests (in some settings); - Professional ethos.	- Pressure, primarily through #27.	- Increased awareness. -Pressure from lobby groups, particularly #1,2 and 4 (in some settings)	Lack of information/ awareness - Lack of interest/time; - Lack of effective political power.
#27 Physician and other health care professional organisations	Represent interests of #26.	- Pressure relevant policy makers and institutions. - Create awareness among members, the public, politicians and policy makers.	- Pressure by members.	- Lack of effective political power.
#28 Patients/ citizens of consumer country states	- Keep costs for medicines low; - Access to antibiotics.	- Support NGO’s; - Vote, exert political pressure; - (When possible) buying “environmentally certified” antibiotics.	- Awareness.	- Lack of information/ awareness; - Lack of interest; -Increased costs for medicines; - Lack of effective political power.
#29 Patient organisations	Represent interests of #28.	- Pressure on county governments or inter-regional coordinating bodies; - Improve awareness.	- Pressure by members;	- Lack of effective political power.
#30 Multinational governing bodies (e.g. the EU)	- Represent member states; - Streamlining the national policies.	- Regulate; - Negotiate, pressure; -Subsidize sustainable practices; -Research funding.	- Political pressure; - Treaties, multilateral agreements, foreign pressure.	- Non-aligning interests between member states; - Lobbying; - Lack of jurisdiction. Outside of e.g. EU.
#31 Agencies of multistate bodies (such as the European Medicines Agency)	- Follow statutes and directives as defined by #14.	- Amend licensing requirements (ERA) to include risks for AMR selection and production emissions; - Include environmental considerations in GMP; - facilitate transparency of production chains.	- Steering by #30.	- Lack of research data to define demands; - Lack of jurisdiction outside of e.g. EU.
#32 Media	- Profit; - Credibility; - Public interest.	- Improve awareness; - Expose polluters; - Demand action from the majority of actors.	- More viewers/readers; - Curiosity; - Increased credibility.	- Opacity of productions chains; -Lack of emission data.
# 33 Scientific researchers and universities	- Reputation; - Receive funding.	- Generate knowledge; - Educate and create awareness among other actors; - Propose scientifically funded actions for e.g. regulation and procurement.	- Curiosity - Reputation; - Funding;	- Institutional barriers to multidisciplinary and/or international cooperation; - Limited access to data and samples from industry.

Additional File 1 and Table 1 are included in the correction article. The original article has been corrected.

## Supplementary information

**Additional file 1.** Relevant actor types and their interests.
