# Supplementary material for: Managing pollution from antibiotics manufacturing: charting actors, incentives and disincentives
Source: Environ Health. 2019 Nov 6;18:95. doi: 10.1186/s12940-019-0531-1 (PMC6833301; doi:10.1186/s12940-019-0531-1)
Supplement: Supplementary file 1 — Additional file 1. Relevant actor types and their interests. [file 12940_2019_531_MOESM1_ESM.pdf]

Additional file 1 (Nijsingh et al; https://doi.org/10.1186/s12940-019-0531-1)

Relevant actor types and their interests

In relation to the problem of industrial antibiotic pollution, we have found 33 different actor types with their own specific types of interests. We do not exclude the possibility that further types, with their own specific types of interests, may be added to the list, but the ones we present here represent a minimum of what needs to be considered in relation to the challenge of industrial antibiotics pollution.

Industry

The pharmaceutical industry has long-term interests in curbing antibiotic resistance - as it threatens to undermine the pharmaceutical market. Also, companies have an interest to be ahead of the policy curve: to influence the timeframes and design of foreseen policies aimed at their behaviour. Furthermore, if industry takes steps to self-regulate before hard policy is introduced, they can be in a better position to accommodate to both regulation and to "green" market competitors. While all industry actors are generally motivated by business considerations and a profit motive, the more precise interests of different types of companies may differ in relevant ways. We should first distinguish between (note, however, that the distinction is not always clear-cut):

1. Research-based pharmaceutical companies. Colloquially referred to as 'Big Pharma'. These are the companies that develop, produce and market patented drugs under a brand name.
2. Generic pharmaceutical companies. These companies produce copies of drugs for which the patent has expired.

Both generic and research-based companies have an interest in a good turnover and reduced costs, but since they have different revenue models, they also have differing specific interests. This relates in large part to the fact that for generic companies, branding will usually matter less than for research-based companies, because the latter rely much more strongly on for example advertising strategies. Thus, while concerns related to public image and reputation may be central for research-based companies, they will likely have less importance for generic companies. Price competition on the other hand is always very strong on the generic market, and only to a lesser extent on the market for drugs whose patent has not yet expired (1). The two company types also differ with regard to their interests in relation to state actors, for example regarding systems for patenting and licensing of drugs, generic substitution schemes for public subsidy, and schemes for controlling and reducing the costs of drug procurement in health systems.

3. Subcontracting pharmaceutical companies. The production of active pharmaceutical ingredients (API's) is often outsourced to subcontractors (2).

API subcontractors have even less interest in maintaining a brand image than generic producers, as the latter still market their own products on an open market, while the former mainly deliver raw material to other companies for further processing. However,

this context of the subcontractor business also means that such companies have a strong interest in maintaining good relationships with generic and research-based companies buying their products. The interests of generic and subcontractor API-producers vis-à-vis state actors also differ significantly from research-based companies, as they usually do not operate in high consumption contexts, but in low- and middle resource settings, such as India and China (3). Their interests are therefore less linked to how institutions responsible for health services act, but may instead be sensitive to the actions of institutions responsible for industry infrastructure, trade and environmental protection. It is important to note that the interests of all three types of pharmaceutical companies are mostly competitive: the interests are about outperforming (or at least not being outperformed by) their market competition. This implies that their willingness to 'play along' with various policy measures will likely depend on what others companies are doing. Therefore, we mention here as a separate category the umbrella organisations and more ad hoc collaborations, whose aim it is to level the playing field and coordinate actions of different pharmaceutical companies:

4. Umbrella organisations/ collaborations between pharmaceutical companies. One example here is the AMR Industry Alliance with "over 100 biotech, diagnostics, generic and research-based pharmaceutical companies and associations" (4) that recently published a pledge to work for more sustainable production (5). The Pharmaceutical Supply Chain Initiative (PSCI) is more established, and hosts over 30 companies and partners (6). Pharmaceutical companies are also united in various coordinating organisations, such as the global IFMPA, the EFPIA (Europe), LIF (Sweden) (7) and the OPPI (India).

The interests of umbrella organisations presumably align with those of their participating members, albeit that the role of competitive advantage is more limited.

Other commercial actors

5. Owners of pharmaceutical companies

Organisations that belong to this actor type have interests that are different from those of the companies they invest in. Owners set the profit expectation for pharmaceutical companies based on financial background preferences, but they may also have other interests of relevance. At least some investors, such as banks and pension funds, may be interested in setting limits to the purposes for which their money is used, for example, regarding sustainability. One reason for this is that they have an interest in maintaining reputation. Additionally, especially pension funds have reasons to consider long-term developments in relation to the interests of their clients. At the same time, more short-term investors may act to restrict the room for corporate responsibility targets of pharmaceutical companies, or press for increased profit expectations that incentivize producers to focus less on environmental safeguards (8).

6. Waste water treatment plants (WWTPs) that clean waste water resulting from manufacturing (9, 10)

Industrial wastewater may be treated by dedicated WWTPs (individual or common to several manufacturers in a small region) or by municipal WWTPs where the industrial waste is mixed with household sewage. The WWTP companies as well as their umbrella organizations (EuREAU in Europe, Swedish Water in Sweden) have an interest to meet set limits for the concentrations of APIs in discharges – but to date this is rarely if ever the case. Since the installation of equipment to effectively treat effluent can be expensive and various methods for handling hazardous pharmaceutical manufacturing waste are possible (11), WWTPs face difficult choices on which treatment is considered sufficient. Depending on how they are funded, it may be challenging to get coverage for such investments and running costs. In doing so, they would need to provide reliable services that continue to be in demand, while still operating a viable business. It may therefore be in their interest to participate in industry-society collaboration to create frameworks, where the WWTPs articulate what waste water should be transported to the WWTP, and provide technical solutions for local or national environmental authorities. Likewise, the WWTPs have interests in providing technical solutions for waste monitoring and treatment commissioned by production plants – as well as scientifically valid norms for measuring environmental contamination.

7. Parallel importers – these are companies that buy drugs in countries where the price is low, in order to repackage and resell them where the price is higher.

As parallel importers basically "piggyback" on independently established pharmaceutical trade arrangements, they have little interest in the details of the production chain, and limited ability to acquire such knowledge. For this reason, a parallel importer recently challenged in court the practice of the Stockholm Country Council in Sweden to ask for environmental information relating to the production chain in their procurement. The main argument was that such procurement criteria limited the free flow of goods across European borders. The parallel importer lost the case (12). In general, their interests with regard to public policy are complex, as one aspect that may improve their business is policy differences between states, for example with regard to environmental emissions or drug licensing, subsidising and procurement by health systems.

State and other public actors in producing countries

8. Producing countries. States in which production is based have options to design, implement and enforce appropriate regulation for producers and WWTPs. We use 'state' and 'country' here more or less interchangeably for the conglomerate of politicians, public servants, political parties, and other public actors on a national level. Producer states also may use their influence on an international level.

The government of a producer state has an interest in maintaining the competitiveness of its businesses, but also to protect the environment and the health of its citizens. All

states have interests to maintain good relations to other states, which may lead a producer state to commit itself to a wider agenda of global public health and environmental sustainability, with implications for antibiotics pollution. Such commitments may also be in a state's interest due to political aims determined domestically. In a long-term perspective, the interest in sustainable antibiotics production is supported from these points of view, but from a shorter perspective they may conflict, especially when the producer state is in strong need of enhanced economic development. This is the typical situation for states housing generic producers and API subcontractors, such as India. Apart from the legislative and executive local and national government, a production state will also house public institutional actors with interests of relevance for industrial antibiotics pollution, for instance:

9. Environmental oversight agencies, such as the Central Pollution Control Board (CPCB) in India are authorized to implement environmental standards, although their actual power may vary.
10. Citizens of producer states. Public opinion may influence both business, political and public institutional action related to antibiotics pollution.
11. Citizen interest groups, environmental and human rights NGOs. For example: SumOfUs, National Green Tribunal (NGT), Health Care Without Harm, European Environmental Bureau, European Public Health Alliance and Health and Environment Alliance. Both the goals and the possibilities to act for these organisations vary widely and their influence is often indirect and difficult to gauge. However, they offer a way for individual citizens to coordinate their actions in order to create more effective pressures on institutional actors (private and public) to attend to shared citizen interests.

International health and trade institutions

International trade is subject to international policy making and agreements.

12. Inter-governmental political forums, such as the G7, the G20, and intergovernmental bodies on global trade, such as the World Trade Organization (WTO), play an important role in setting the institutional background for the global situation with regard to the flow of drugs and API's.
13. United Nations agencies, such as the World Intellectual Property Organisation (WIPO), the United Nations Development Programme (UNDP), the United Nations Environment Programme (UNEP), and the World Health Organisation (WHO), have interests both regarding the global landscape of drug trade, and/or global health policy. Of these agencies, the WHO has already adopted a significant role in the global agenda setting on antibiotic resistance, promoting prevention and surveillance, as well as and guiding national policy. The WHO

moreover has legal authority to serve as a facilitating platform for international treaty making efforts (13).

All of these organizations have common interests on global coordination and facilitation. However,their exact role varies. While the WHO is focused on health policy and related infrastructure, the WTO's mission concentrates on global trade agreements. Such different interests may easily pull in opposite directions in areas related to health and the environment, as measures taken to target particular parties that are of special concern from a health or environmental perspective may be seen as illegitimate trade restrictions. However, they may also enforce actions to ensure fair business competition that may serve to make other policy measures easier to accept by commercial parties.

State and other public actors in consumer countries

14. Consumer countries have possibilities to implement regulation and use financial and other incentives to motivate commercial actors within the country. They may also apply pressure to other consumer and producing countries. In the Swedish case, the twenty county governments play an important role, each with its own directly elected political representatives, its own tax collection for healthcare purposes and with autonomy to make decisions within the boundaries of national law. In federative states, such as Germany or the USA, the responsibility may instead be placed at the state level, in between the federal government and counties/municipalities within states that are members of the federation.

Just as in the case of producer states, a number of relevant public institutional actors operate at the national level of consumer states.

15. Licensing agencies, such as the Medical Products Agency (*Läkemedelsverket*, LV) in Sweden, are responsible for the standards and licensing of medical products. In Sweden, LV also has power to decide which drugs are 'interchangeable' within the national subsidy system (14). While some drugs entering the European market do this only via EMA procedures, most drugs currently available were licenzed on a national level (15).
16. Agencies that govern the use of public money to subsidize drugs, in Sweden, the Dental and Pharmaceutical Benefits Agency (*Tandvårds- och läkemedelsförmånsverket*, TLV).

17. Agencies or other national professional bodies formulating and overseeing directives for drug prescription in the form of standardized treatment, in Sweden divided between the two agencies, the National Board of Health and Welfare (*Socialstyrelsen*, SoS), and The Healthcare Inspectorate (*Inspektionen för vård och omsorg*, IVO).

18. Public health agencies, for example, The Public Health Agency of Sweden (*Folkhälsomyndigheten*, FHM) has a specific charter for coordinating and oversee actions to manage antibiotic resistance.

19. Agencies that offer support and guidance for the public procurement of drugs, in Sweden, The National Agency for Public Procurement (*Upphandlingsmyndigheten*).

While few of these agencies are currently involved actively in actions in response to industrial antibiotic pollution, their respective interests (based on their charters) in minding about health, good health policy and the quality of health systems can be related to the antibiotic resistance challenge posed by this pollution in various ways. Based on that interest, they may also find reasons to take actions to influence the situation regarding industrial antibiotic pollution.

20. Public hospitals and clinics are important buyers and prescribers of antibiotics, but also subcontract to privately owned hospitals and clinics to have them deliver publicly funded health services.

21. In the case of Sweden, the county governments own and operate public hospitals and primary care clinics. Also, regional medical products committees (*Läkemedelskommittér*), are connected to the county policy making and issue directions for prescription practice through "recommendation lists", which affect prescriptions by all doctors in the county and also applies to use outside of hospitals (16).

22. Decisions regarding prescription guidance and rights, priority setting and procurement may vary between different counties. For this reason, a nation state or the regional governments in collaboration often set up inter-regional coordinating bodies for priority-setting, procurement recommendations and price negotiations with drug companies so that common standards and equal conditions apply across regions. In Sweden, this is a rather recent development, and is exemplified by *NT-rådet*, a policy guiding committee run by the collaborative organization of counties and municipalities (*Sveriges kommuner och landsting*, SKL) (17).

All of these public actors at the regional or interregional level share interests in attaining affordable drugs, as well as in delivering good quality of care, which may give reasons for action in relation to antibiotic resistance.

Commercial actors in consumer countries

23. Privately funded and operated clinics and hospitals in Sweden should be considered as commercial buyers of antibiotics.

24. Pharmacies, however, buy the bulk of antibiotics in Sweden. While the total of purchases made by pharmacies was 28, 4 billion Swedish Krona in 2015, the remaining procurements made by County Councils amounted to 7,5. (18)

25. Insurance companies. In states where the health system is less publicly funded than in the Swedish case, private insurance companies replace the importance of public regional operators of health systems proportionally to the extent to which these systems are privately rather than publicly funded.

While operating in a landscape of business rather than public service, these actors nevertheless share the interest with state and other public actors to curb pharmaceutical costs. Rather than quality of health services, however, their foremost other interest will be that of customer satisfaction related to continued prospects for good business. While this interest may overlap with an interest in quality of healthcare services, this is not necessarily so, as customer satisfaction may depend on many factors. In addition, a business need not have the long-term perspective of a public service (as its interests are determined by the investment preferences of its owners), and thus have a weaker interest in keeping its supply of service sustainable.

Professional and private actors and organisations

Typically, a number of individual and informally organized actor types are of importance for what actions are taken within consumer states that may have bearing on the problem of industrial antibiotics pollution.

26. Physicians and other health care professionals. Health care professionals have an explicit interest in contributing to a sustainable health care system. At the same time, however, they typically have limited time to spend on each patient, and taking the environmental impact of various products into account may come at the cost of spending resources on other priorities.

27. Physician and other health care professional organisations, as well as other medical organizations lobby at all levels, as well as coordinate professional standards of good practice. In Sweden, these are the Swedish Medical Association (*Läkarförbundet*), and the Swedish Society of Medicine (*Svenska läkaresällskapet*).

28. Patients. Physicians and patients share a long-term interest in avoiding ABR to undermine the effectiveness of healthcare, as well of course in successful treatment of the patient. Still, with regards to antibiotic treatment, the interests of physicians may in some cases conflict with those of patients, given the expected stronger public health engagement of the physician, and the stronger self-interest of the patient. In the case of antibiotic pollution, however, there should be no conflict as the choice of options would not be about choosing a less effective treatment, but to use a product associated with less pollution during its manufacturing.

29. Patient organisations may (and often do) put pressure on county governments or inter-regional coordinating bodies. Their main interest is to represent the interests of #28.

Multi-national consumer state actors

The behaviour of both consumer and producing states can only be properly understood in the context of global diplomacy and multinational institutions.

30. Multinational governing bodies, such as the European Union. The relevant legislative and executive European institutions, that is the European Parliament, the Council of Ministers, and the European Commission, have a stake in the global market in medicine, not in the least because they represent a population that consumes over twenty percent of the global drug sales (19). The European institutions aim to promote the interests of the EU's member states, but also have a distinct interest in harmonizing standards and rules throughout the EU. In general, these institutions do not want to impose stronger demands on internal EU producers than on producers outside of EU as this would lead to market disadvantages for the EU based companies.

31. Agencies of multistate bodies may, at the same time take on distinct roles of themselves. Decentralized European agencies are organisations distinct from the mentioned European governing institutions, set up to perform various specific tasks. In the case of the EU, three agencies are of particular relevance for industrial antibiotics pollution. First, the European Medicines Agency (EMA) is a scientific agency which aims to harmonise safety, assessment and oversight standards regarding drugs in different EU countries (20). The European Environment Agency (EEA), has as its main task to provide information, for example regarding environmental risk, underlying central policy decisions in this area, such as the Water Framework Directive which was first drafted by the European Commission in 2000 (21, 2267).

Other actors

Two types of actors need to be mentioned that may interact with and influence, as well as be influenced by, all of the earlier mentioned actors, while remaining to a large extent independent of them.

32. Media, that is journalism in the broad sense, such as newspapers, broadcasting (today via numerous pathways and increasingly online), blogs and so on, play an important role in information and opinion to actors on various levels (23, 24, 25, 26).

Media actors' interests are usually bound up with an agenda of journalistic ideals, for example regarding the value of the expected width and power of impact of information relayed. However, single media actors may also act in collaboration with any of the other ones, helping to empower these in the pursuit of their respective interests. Sometimes this leads to conflicts of interests debated in the area of media ethics.

33. Scientific researchers and universities provide data on the impact and extent of pollution, on responsible discharge limits and waste water treatment, assessing risks of emergence and transmission of resistance, as well social conditions for change and economic risk assessments, to name but a few relevant areas. Scientists play an important role in the extent to which they may afford both the monitoring of industrial antibiotics pollution, holding to account polluters and other responsible actors, and societal development to manage this problem.

Like the media, researchers and their institutions are ideally guided by interests coming out of their own internal value system with regard to systematic and critical pursuit of qualified knowledge. However, just as the media, researchers may mix this interest with aims to assist other actors, and thereby expose themselves to possible conflicts of a sort debated in terms of research ethics and integrity.

References

1. MDTV Alliance (2018). API Industry Guide: http://www.mdtvalliance.org/top-api-manufacturers. Accessed on 27 Mar 2019.
2. Bengtsson-Palme J, Kristiansson E, Larsson DJ. Environmental factors influencing the development and spread of antibiotic resistance. FEMS microbiology reviews. 2017 Oct 24;42(1): https://doi.org/10.1093/femsre/fux053.

3. Van Arum, Patricia (2013). Fine-Chemical Producers and Contract API Manufacturers Expand: http://www.pharmtech.com/fine-chemical-producers-and-contract-api-manufacturers-expand. Accessed on 27 Mar 2019.

4. AMR Industry Alliance. https://www.amrindustryalliance.org. Accessed on 22 Mar 2019.

5. AMR Industry Alliance, manufacturing & environment: https://www.amrindustryalliance.org/shared-goals/common-antibiotic-manufacturing-framework. Accessed on 27 Mar 2019.

6. Pharmaceutical supply chain initiative: https://pscinitiative.org. Accessed on 27 Mar 2019.

7. LIF (Swedish Association of the Pharmaceutical Industry). Ethical Rules For The Pharmaceutical Industry In Sweden (2017). https://www.lif.se/globalassets/etik/dokument/ler-english-20180212-rp.pdf. Accessed on 22 Mar 2019.
8. Schwartz MS, Saiia D. Should Firms Go "Beyond Profits"? Milton Friedman versus Broad CSR 1. Business and Society Review. 2012 Mar;117(1):1-31.
9. Ågerstrand M, Berg C, Björlenius B, Breitholtz M, Brunström B, Fick J, Gunnarsson L, Larsson DJ, Sumpter JP, Tysklind M, Rudén C. Improving environmental risk assessment of human pharmaceuticals. Environmental Science & Technology. 2015 Apr 16;49(9):5336-45.
10. U.S. Centers for Disease Control and Prevention, Initiatives for Addressing Antimicrobial Resistance in the Environment Current Situation and Challenges: https://wellcome.ac.uk/sites/default/files/antimicrobial-resistance-environment-report.pdf. Accessed on 24 Apr 2019.
11. Larsson DJ. Pollution from drug manufacturing: review and perspectives. Philosophical Transactions of the Royal Society B: Biological Sciences. 2014 Nov 19;369(1656):20130571.
12. Sågänger, J. Parallellhandelsföretag utmanar SLL:s miljökrav i domstol (8 Mar 2018): https://www.insiktmedicin.se/articles/515305/2018-03-08-12-07-24-parallellhandelsforetag-utmanar-sll-s-miljokrav-i-domstol. Accessed on 26 April 2019.
13. Taylor AL. Making the World Health Organization work: a legal framework for universal access to the conditions for health. Am. JL & Med.. 1992;18:301.
14. Läkemedelsverket, Utbytbara läkemedel: https://lakemedelsverket.se/malgrupp/Halso---sjukvard/Forskrivning/Utbytbara-lakemedel-. Accessed on 27 Mar 2019.
15. European Medicines Agency, Authorisation of Medicines: http://www.ema.europa.eu/ema/index.jsp?curl=pages/about_us/general/general_content_000109.jsp. Accessed on 22 Mar 2019.
16. Janusinfo, Kloka listan: https://www.janusinfo.se/download/18.10adba9e1616f8edbc9c690/1535626533580/Kloka-Listan-2018.pdf. Accessed on 27 Mar 2019.

17. Sveriges Kommuner och Landsting, Landstingens samverkansmodell för läkemedel: https://skl.se/halsasjukvard/lakemedel/samverkansmodelllakemedel.2109.html. Accessed on 27 Mar 2019.
18. SEPA, Procurement of pharmaceuticals in an environmental context and its inclusion into the CSR Compass (2016): http://www.swedishepa.se/upload/miljoarbete-i-samhallet/miljoarbete-i-eu/Procurement-of-pharmaceuticals-in-an-environmental-context-and-its-inclusion-into-the-CSR-Compass-2016.pdf. Accessed on 27 Mar 2019.
19. European federation of pharmaceutical industries and associations (efpia), The Pharmaceutical Industry in Figures: https://www.efpia.eu/media/219735/efpia-pharmafigures2017_statisticbroch_v04-final.pdf. Accessed on 27 Mar 2019.
20. Pharmaceuticals in the environment http://ec.europa.eu/environment/water/water-framework/info/intro_en.htm. Accessed on 27 Mar 2019.
21. Läkemedelsverket (2018). Läkemedel i miljön. Report Dnr: 4.3.1-2018-048910. Uppsala: Läkemedelsverket.
22. European commission, Priority substances supporting information and documentation: http://ec.europa.eu/environment/water/water-dangersub/lib_pri_substances.htm. Accessed on 27 Mar 2019
23. VICE News, Superbugs: The Dark Side of India’s Drug Boom: https://www.youtube.com/watch?v=ASBeOZlujSw. Accessed on 27 Mar 2019.
24. Brennpunkt, Bakterietrusselen: https://tv.nrk.no/serie/brennpunkt/2016/MDDP11000416. Accessed on 24 Apr 2019.
25. Litovsky, Alejandro, Antibiotic waste is polluting India and China's rivers; big pharma must act (Oct. 2016): https://www.theguardian.com/sustainable-business/2016/oct/25/antibiotic-waste-pollution-india-china-rivers-big-pharma-superbugs-resistance. Accessed on 27 Mar 2019.
26. Scherman, Lena, Läkemedelsutsläpp gör människor sjuka (30 May 2010): https://www.svt.se/nyheter/vetenskap/lakemedelsutslapp-gor-manniskor-sjuka. Accessed on 27 Mar 2019.
